# Deontological guilt feelings in eating disorders: data from an Italian experience

**DOI:** 10.1192/j.eurpsy.2025.1406

**Published:** 2025-08-26

**Authors:** F. Raffone, D. Atripaldi, F. Mancini, A. M. Saliani, A. M. Monteleone

**Affiliations:** 1 Department of Mental Health, Asl Napoli 1 Centro, Università degli Studi della Campania “L. Vanvitelli”; 2 Department of Advanced Medical and Surgical Sciences, Università degli Studi della Campania “L. Vanvitelli”, Naples; 3 Scuole di Psicoterapia Cognitiva APC-AIPC-SPC-SICC, Rome; 4 Department of Mental Health, Università degli Studi della Campania “L. Vanvitelli”, Naples, Italy

## Abstract

**Introduction:**

Guilt and shame are common emotional experiences that may influence the prognosis and treatment of many psychiatric disorders.

**Objectives:**

The aim of this study was to examine the role of guilt and shame in individuals with eating disorders (ED).

**Methods:**

Forty-three adults diagnosed with anorexia nervosa, bulimia nervosa, and binge eating disorder were included in the study. They completed the following questionnaires: the Moral Orientation Guilt Scale (MOGS), which measures different components of guilt, and the Eating Disorder Inventory 2 (EDI-2), which measures ED psychopathology. To assess the relationships between MOGS and EDI-2 subscales, Spearman’s correlations and a stepwise multiple regression have been conducted including all patients in a unique ED group.

**Results:**

Positive correlations were found between the EDI-2 bulimia subscale and the MNV (moral normal violation) subscale of the MOGS (0.26, p.=0.05), between the EDI-2 interpersonal distrust subscale and the MNV subscale of the MOGS (Rho=0.28, p=0.03), and between the EDI-2 interpersonal distrust subscale and both altruistic guilt components of the MOGS (Rho=0.33, p=0.01 for harm; Rho=0.29, p=0.03 for empathy). The multiple linear regression model was significant (R²=0.29, F=8.38, p.<0.01) and showed age (t=-2.9, p<0.01) and the HARM subscale (t=3.4, p<0.01) as predictors of interpersonal distrust.

**Conclusions:**

The results provide preliminary evidence for a possible role of guilt in the aetiopathogenesis of ED. Sensitivity to altruistic guilt, and especially to the harm caused, seems to influence the ability to trust others. Avoidance, distancing, or closure may be strategies to overcome high sensitivity to guilt. Further studies with larger samples, including both ED patients and healthy individuals, are needed to determine the role of guilt in EDs.

**Disclosure of Interest:**

None Declared

